# Acceleration of tooth movement during orthodontic treatment - a frontier in Orthodontics

**DOI:** 10.1186/2196-1042-14-42

**Published:** 2013-10-29

**Authors:** Ghada Nimeri, Chung H Kau, Nadia S Abou-Kheir, Rachel Corona

**Affiliations:** Department of Orthodontics, University of Alabama, 305 School of Dentistry Building, 1919 7th Avenue south, Birmingham, AL 35294-0007 USA

**Keywords:** Accelerating tooth movement, Biology, Photobiomodulation

## Abstract

Nowadays, there is an increased tendency for researches to focus on accelerating methods for tooth movement due to the huge demand for adults for a shorter orthodontic treatment time. Unfortunately, long orthodontic treatment time poses several disadvantages like higher predisposition to caries, gingival recession, and root resorption. This increases the demand to find the best method to increase tooth movement with the least possible disadvantages. The purpose of this study is to view the successful approaches in tooth movement and to highlight the newest technique in tooth movement. A total of 74 articles were reviewed in tooth movement and related discipline from 1959 to 2013. There is a high amount of researches done on the biological method for tooth movement; unfortunately, the majority of them were done on animals. Cytokine, PTH, vitamin D, and RANKL/RANK/OPG show promising results; on the other hand, relaxin does not accelerate tooth movement, but increases the tooth mobility. Low-level laser therapy has shown positive outcome, but further investigation should be done for the best energy and duration to achieve the highest success rate. Surgical approach has the most predictable outcomes but with limited application due to its aggressiveness. Piezocision technique is considered one of the best surgical approaches because it poses good periodontal tissue response and excellent aesthetic outcome. Due to the advantages and disadvantages of each approach, further investigations should be done to determine the best method to accelerate tooth movement.

## Review

### Introduction

Orthodontics has been developing greatly in achieving the desired results both clinically and technically. This is especially so by using new technologies, like stimulation software that can assist in treatment planning and translational products. In addition, continuous modification of wires and brackets as a result of the biomechanical efficiencies in orthodontics has greatly improved. However, these biomechanical systems may have reached their limit and there is a need to develop new methods to accelerate teeth movement.

Today, it is still very challenging to reduce the duration of orthodontic treatments. It is one of the common deterents that faces orthodontist and causes irritation among adults plus increasing risks of caries, gingival recession, and root resorption.

A number of attempts have been made to create different approaches both preclinically and clinically in order to achieve quicker results, but still there are a lot of uncertainties and unanswered questions towards most of these techniques. Most attempts can broadly be categorized into biological, physical, biomechanical, and surgical approaches. Before going into details of these attempts, we need to understand the basics of orthodontic tooth movements and the factors that initiate inhibition and delayed tooth movement.

Orthodontic tooth movement occurs in the presence of a mechanical stimuli sequenced by remodeling of the alveolar bone and periodontal ligament (PDL). Bone remodeling is a process of both bone resorption on the pressure site and bone formation on the tension site [1]. Orthodontic tooth movement can be controlled by the size of the applied force and the biological responses from the PDL [2]. The force applied on the teeth will cause changes in the microenvironment around the PDL due to alterations of blood flow, leading to the secretion of different inflammatory mediators such as cytokines, growth factors, neurotransmitters, colony-stimulating factors, and arachidonic acid metabolites. As a result of these secretions, remodeling of the bone occurs [3, 4].

### Methods of accelerating tooth movement

There are three phases of tooth movement: the initial phase, which is characterized by rapid movement after the application of force; followed by a lag period, where little or no movement, and the last phase, where gradual or sudden increase of movement occurs [5].

The early phase of tooth movement involves acute inflammatory responses characterized by leucocytes migrating out of blood capillaries and producing cytokines, which stimulates the excretion of prostaglandins and growth factors [6]. The acute phase is followed by the chronic phase that involves the proliferation of fibroblast, endothelial cells, osteoblasts, and alveolar bone marrow cells remodeling process [4].

#### Biological approach

Experiments have been done using these molecules exogenously to enhance tooth movement both in animal experiments and humans. Example of these molecules are prostaglandin E (PGE), cytokines that include lymphocytes and monocytes-derived factors, receptor activator of nuclear factor kappa B ligand (RANKL), and macrophage colony-stimulating factor (MCSF) [7–9] (Table 1).

**Table 1:** **Biological approaches to enhance tooth movement**

Authors	Biological molecules tested	Animal or humans	Duration	Acceleration
Saito et al. [9]	PGs and IL-1	Cats	Weeks	Yes
Yamasaki et al. [10]	PGs	Rats	Weeks	Yes
Yamasaki et al. [11]	PGs	Monkeys	Weeks	Yes
Leiker et al. [7]	PGs	Rats	Weeks	Yes
Yamasaki et al. [12]	PGs	Human	Months	Yes
Seifi et al. [13]	PGs + Ca	Rats	Weeks	Yes and stabilize root resorption
Seifi et al. [13]	PGs − Ca	Rats	Weeks	Yes
Kanzaki et al. [14]	RANKL/RANK	Animals	Weeks	Yes
	OPG	Animals	Weeks	Yes
Nishijima et al. [15]	RANKL/RANK/OPG and root resorption	Human	Months	Relation with root resorption
Collins et al. [16]	Vitamin D	Cats	Weeks	Yes
Kale et al. [17]	Vitamin D and PGs	Rats	Weeks	Yes
Soma et al. [18]	PTH	Rats	Weeks	Yes
Soma et al. [19]	PTH	Rats	Weeks	Yes
Liu Zi et al. [20]	Relaxin	Rats	Weeks	Yes
Madan et al. [21]	Relaxin	Rats	Weeks	Effect on collage fibers
Mcgorray et al. [22]	Relaxin	Human	Weeks	No

PGs, prostaglandins; RANKL, receptor activator of nuclear factor kappa B ligand; PTH, parathyroid hormone; Ca ,Calcium.

##### Effect of cytokines on tooth movement

High concentration of cytokines such as interleukins IL-1, IL-2, IL-3 IL-6, IL-8, and tumor necrosis factor alpha (TNF) were found to play a major role in bone remodeling; moreover, interleukin-1 (IL-1) stimulates osteoclast function through its receptor on osteoclasts [3]. It was also found that mechanical stress due to orthodontic treatment increased the production of prostaglandin PGE and IL-1 beta in the periodontal ligaments. These experiments were done on cats where one canine was tipped distally by 80 g of force from hours to days, then immunohistochemistry and microphotometry experiments where done to measure the intensity of PGE and IL-1 beta which was found to be highest on the tension [9].

Other cytokines which are also involved in the acceleration of tooth movement are RANKL, which is a membrane-bound protein on the osteoblasts that bind to the RANK on the osteoclasts and causes osteoclastogenesis [23–25]. On the other hand, osteoprotegerin (OPG) competes with RANKL in binding to osteoclast to inhibit osteoclastogenesis. The process of bone remodeling is a balance between (RANKL-RANK) system and OPG compound [26, 27]. In relation to this, using biological molecules in the acceleration of tooth movement [14] has been shown in two unique experiments in which it was demonstrated that the transfer of RANKL gene to the periodontal tissue induced prolonged gene expression for the enhancement of osteoclastogenesis and acceleration of tooth movements in rats. On the other hand, the transfer of OPG gene inhibited orthodontic tooth movements [28]. In another study it was found that juvenile teeth move faster than adults, which is due to the lower amount of RANKL/OPG ratio in the gingival crevicular fluid (GCF) in adult patients measured by the enzyme-linked immunosorbent assay method.

Also a correlation was found among RANK, OPG, and root resorption during orthodontic teeth movement, and patients with root resorption produced a large amount of RANKL in the compressed site [15, 29].

##### Prostaglandin effect on tooth movement

Prostaglandins (PGs) are inflammatory mediator and a paracrine hormone that acts on nearby cells; it stimulates bone resorption by increasing directly the number of osteoclasts. *In vivo* and *in vitro* experiments were conducted to show clearly the relation between PGs, applied forces, and the acceleration of tooth movement. Yamasaki [10, 11] was among the first to investigate the effect of local administration of prostaglandin on rats and monkeys. In addition, experiments done in [7] have shown that injections of exogenous PGE2 over an extended period of time caused acceleration of tooth movements in rats. Furthermore, the acceleration rate was not affected by single or multiple injections or between different concentrations of the injected PGE2. However, root resorption was very clearly related to the different concentrations and number of injections given. It has also been shown that the administration of PGE2 in the presence of calcium stabilizes root resorption while accelerating tooth movement [13].

Furthermore, chemically produced PGE2 has been studied in human trials with split-mouth experiments in the first premolar extraction cases. In these experiments the rate of distal retraction of canines was 1.6-fold faster than the control side [12].

##### Effect of Vitamin D3 on tooth movement

Vitamin D3 has also attracted the attention of some scientist to its role in the acceleration of tooth movement; 1,25 dihydroxycholecalciferol is a hormonal form of vitamin D and plays an important role in calcium homeostasis with calcitonin and parathyroid hormone (PTH).

Another set of investigators [16] has made an experiment where they have injected vitamin D metabolite on the PDL of cats for several weeks; it was found that vitamin D had accelerated tooth movement at 60% more than the control group due to the increasement of osteoclasts on the pressure site as detected histologically. A comparison between local injection of vitamin D and PGEs on two different groups of rats was also investigated. It was found that there is no significant difference in acceleration between the two groups. However, the number of osteoblasts on the pressure side which was injected by vitamin D was greater than on the PGE2 side. This indicates that vitamin D may be more effective in bone turnover [17].

##### PTH effect on tooth movement

PTH has been shown to accelerate orthodontic tooth movement on rats, which was studied by continuous infusion of PTH (1 to 10 μg/100 g of body weight/day) implantation in the dorsocervical region, and the molars were moved 2- to 3-fold faster mesially by orthodontic coil spring [18].

Some studies have shown that locally injected PTH induces local bone resorption, and it is more advantageous to give PTH locally rather than systemically [30]. The development of a slow-release application that keeps the local concentration of PTH for a long time was very efficient as shown later in [19] where the daily injection of PTH dissolved in gel medium allowed a slow release which caused 1.6-fold faster acceleration of teeth compared to daily injection of PTH dissolved in saline solution which did not cause any acceleration.

##### Relaxin effect on tooth movement

Relaxin effect has also been investigated. Relaxin is a hormone that helps during childbirth by widening of the pubic ligaments in females and is suggested to be present in cranial suture and PDL [31]. The role of relaxin is known in the remodeling of soft tissue rather than remodeling of bone. It has been shown that it increases collagen in the tension site and decreases it in compression site during orthodontic movement [32, 33]. Also, the administration of human relaxin may accelerate the early stages of orthodontic tooth movement in rat experiments [20]. However, another study showed that human relaxin does not accelerate orthodontic tooth movement in rats, but can reduce the level of PDL organization and mechanical strength of PDL and increase tooth mobility [21]. In these experiments *in vitro* studies were also performed to test the PDL mechanical strength and tooth mobility using tissue from additional 20 rats that had previously received the same relaxin treatment for several days [21].

The remodeling of PDL by relaxin might reduce the rate of relapse after orthodontic treatment as suggested by others [34]. Recently, randomized clinical trials on humans were done by weekly injections of 50 μg of relaxin or placebo control for 8 weeks. Tooth movement was measured weekly on polyvinyl siloxane impressions which were scanned digitally. There was no significant difference between the relaxin and the placebo control group regarding the acceleration and relapse [22]. However, the mechanism of how relaxin accelerates tooth movement is not yet fully understood.

#### Device-assisted treatment

Another approach in accelerating tooth movement is by using device-assisted therapy (Table 2). This technique includes direct electric currents, pulsed electromagnetic field, static magnetic field, resonance vibration, and low-level laser which was mostly investigated and gave the most promising results.

**Table 2:** **Device-assisted treatment techniques and their effect on tooth movement**

Author	Physical approach used	Rate	Animal/human	Acceleration
Nishimura [35]	Vibrational stimulation	60 Hz, 1.0 m/s (2/8 min/day)	Rats	Yes
Kau et al. [36]	Resonance vibration	20 to 30 Hz/20 min/day	Human	Yes
Davidovitch [37]	Direct electrical current	7 V	Animal	Yes
Fujita et al. 2008 [38]	Low-level laser	810-nm Ga-Al-As diode laser and continuous waves at 100 mW	Rats	Yes
Kawasaki [39]	Low-level laser	830-nm Ga-Al-As diode laser and continuous waves at 100 mW	Rats	Yes
Limpanichkul [40]	Low-level laser	860-nm Ga-Al-As diode and continuous waves at 100 mW	Human	No
Kau [41]	Low-level laser	850-nm LED and continuous wave 60 mW	Human	Yes
Doshi-Mehta G [42]	Low-level laser	800-nm Ga-Al-As diode laser and continuous wave 0.25 mW	Human	Yes

LED, Light-Emitting Diode

The concept of using physical approaches came from the idea that applying orthodontic forces causes bone bending (bone bending theory) and bioelectrical potential develops. The concave site will be negatively charged attracting osteoblasts and the convex site will be positively charged attracting osteoclasts as detected by Zengo [43] in his measurements on dog alveolar bone.

The bioelectrical potential is created when there is application of discontinuous forces, which leads to the idea of trying cyclic forces and vibrations. It has been found that applying vibrations for different duration per day accelerated tooth movements between 15% and 30% in animal experiments [35, 44].

##### Cyclical force device effect on tooth movement

We have also used this concept by using the cyclical force device with patients and achieved 2 to 3 mm/month of tooth movement. The vibration rate was 20 to 30 Hz and used for 20 min/day [36]. Further results needed to be investigated to clearly identify the range of Hertz that can be used in these experiments to get the maximum desired results.

##### Direct electric current effect on tooth movement

Another approach is to use direct electric current. This technique was tested only on animals by applying direct current to the anode at the pressure sites and cathode at the tension sites (by 7 V), thus, generating local responses and acceleration of bone remodeling as shown by group of investigators [37]. Their studies were more successful than the previous attempts because electrodes were placed as close as possible to the moving tooth. The bulkiness of the devices and the source of electricity made it difficult to be tested clinically. Several attempts were made to develop biocatalytic fuel cells to generate electricity intraorally by the use of enzymes and glucose as fuel [45, 46]. Further development of the direct electric device and the biocatalytic fuel cells is needed to be done so that these can be tested clinically.

##### Low-level laser therapy

Photobiomodulation or low-level laser therapy (LLLT) is one of the most promising approaches today. Laser has a biostimulatory effect on bone regeneration, which has been shown in the midpalatal suture during rapid palatal expansion [47], and also stimulates bone regeneration after bone fractures and extraction site [48, 49]. It has been found that laser light stimulates the proliferation of osteoclast, osteoblast, and fibroblasts, and thereby affects bone remodeling and accelerates tooth movement. The mechanism involved in the acceleration of tooth movement is by the production of ATP and activation of cytochrome C, as shown in [38, 50, 51] that low-energy laser irradiation enhanced the velocity of tooth movement via RANK/RANKL and the macrophage colony-stimulating factor and its receptor expression.

Animal experiments have shown that low-level laser can accelerate tooth movement. Furthermore, clinical trial attempts were made in which different intensities of laser were used and different results were obtained [40, 42]. Low-level laser therapy can be a very useful technique for acceleration of tooth movement since it increases bone remodeling without side effects to the periodontium. Laser wavelength of 800 nm and output power of 0.25 mW have indicated significant stimulation of bone metabolism, rapid ossification [39, 49], and also acceleration of tooth movement to 1.5-fold in rat experiments. Lately in a clinical trial study, the laser wavelength they have used in a continuous wave mode at 800 nm, with an output of 0.25 mW, and exposure of 10 s was found to accelerate tooth movement at 1.3-fold higher than the control [42]. In another study done by Kau [41] on 90 subjects (73 test subjects and 17 controls), there was 1.12-mm change per week in the test subjects versus 0.49 mm in the control group. Having said this, there are a lot of contradictory results related to the LLLT. Therefore, more experiments are needed to differentiate the optimum energy, wavelength, and the optimum duration for usage.

#### Surgical approach

The surgical technique has been documented in many case reports. It is a clinically effective technique used for adult patients, where duration of orthodontic treatment may be critical in selected groups of patients. The PDL and alveolar bone remodeling are the important parameters in tooth movement, and bone turnover is known to increase after bone grafting, fracture, and osteotomy.

Several surgical approaches that have been tried in order to accelerate tooth movement were interseptal alveolar surgery, osteotomy, corticotomy, and Piezocision technique (Table 3).

**Table 3:** **Surgical approaches to enhance tooth movement**

Author	Surgical approach used	Animal/Human	Acceleration
Liou [52]	Distraction of the PDL aided by alveolar surgery undermining the interseptal bone	Human	Yes
Ren [53]	Intraseptal alveolar surgery	Dog	Yes
Sukurica et al. 2007 [54]	Rapid canine distalization by segmental alveolar distraction	Human	Yes
Kisnisci [55]	Rapid canine distalization by segmental alveolar distraction	Human	Yes
Iseri [56]	Rapid canine distalization by segmental alveolar distraction	Human	Yes
Sayin [57]	Rapid canine distalization by segmental alveolar distraction	Human	Yes
Lee [58]	Corticotomy-assisted tooth movement	Rats	Not statistically significant
Wilcko et al. 2001 [59]	Accelerated osteogenic orthodontics	Human	Yes
Baloul [60]	Corticotomy	Rats	Yes
Aboul et al. 2011 [61]	Corticotomy	Human	Yes
Han [62]	Intraseptal alveolar surgery	Dog	Yes
Dibart [63]	Piezocision technique	Human	Yes
Hassan [64]	Piezocision technique	Human	Yes
Keser and Dibart 2011 [65]	Piezocision-assisted Invisalign treatment	Human	Yes

##### Interseptal alveolar surgery

Interseptal alveolar surgery or distraction osteogenesis is divided into distraction of PDL or distraction of the dentoalveolar bone; example of both is the rapid canine distraction. The concept of distraction osteogenesis came from the early studies [66] of limb lengthening. Also from surgical treatments of craniofacial skeletal dysplasia, this concept was later adapted in relation to the rapid tooth movement.

In the rapid canine distraction of PDL, the interseptal bone distal to the canine is undermined surgically at the same time of extraction of the first premolars, thus, this will reduce the resistance on the pressure site. In this concept the compact bone is replaced by the woven bone, and tooth movement is easier and quicker due to reduced resistance of the bone [52]. It was found that these rapid movements are during the initial phases of tooth movement especially in the first week as show in [53].

In this technique the interseptal bone is undermined 1 to 1.5 mm in thickness distal to the canine after the extraction of the first premolar, and the socket is deepened by a round bur to the length of the canine. The retraction of the canine is done by the activation of an intraoral device directly after the surgery. It has been shown that it took 3 weeks to achieve 6 to 7 mm of full retraction of the canine to the socket of the extracted first premolars [52].

Rapid canine distraction of the dentoalveolar bone is done by the same principle of the distraction of PDL, with the addition of more dissection and osteotomies performed at the vestibule as shown in [54–57, 63].

In all the studies done, both techniques accelerated tooth movement with no evidence of significant root resorption, ankylosis, and root fracture. However, there were contradictory results regarding of the electrical vitality test of the retracted canines. Liou [52] reported 9 out of 26 teeth showed positive vitality, while Sukurica [54] reported that 7 out of 20 showed positive vitality after the sixth month of retraction. So there are still some uncertainties regarding this technique.

##### Corticotomy and osteotomy

Osteotomy and corticotomy are also surgical techniques that have been clinically used for many years. Osteotomy is when a segment of the bone is cut into the medullary bone and is separated and then moved as a unit as shown in [58, 67].

Corticotomy is one of the surgical procedures that is commonly used in which only the cortical bone is cut and perforated but not the medullary bone, suggesting that this will reduce the resistance of the cortical bone and accelerate tooth movements. It was first tried in orthodontics by Kole [68], where tooth movements were achieved between 6 and 12 months. The technique was further used by others, for example, Grenerson [69] who used this for open bites treatments, and others in [70–72].

In 2001 Wilcko [59] reported that the acceleration of tooth movement is not due to the bony block movement as postulated by Kole [68]; it was rather a process of bone remodeling at the surgical site, which was called regional acceleratory phenomenon (RAP). He developed patent techniques which were called accelerated osteogenic orthodontics (AOO) and periodontal accelerated osteogenic orthodontics. Also, modification of RAP was done by adding bioabsorbable grafting material over the injured bone to enhance healing.

This technique is reported to have postoperative stability and improved retention as shown in [73], but more studies are still needed to be done. The negativity of these surgical techniques is their invasiveness and the acceleration was only in the first 3 to 4 months and it declines with time to the same level of the controls, as shown by others [60–62].

##### Piezocision technique

One of the latest techniques in accelerating tooth movement is the Piezocision technique. Dibart [63] was among the first to apply the Piezocision technique which starts with primary incision placed on the buccal gingiva followed by incisions by Piezo surgical knife to the buccal cortex [74]. Piezocision technique did not cause any periodontal damage as reported by Hassan [64]. Another benefit of this technique is that it can be used with Invisalign, which leads to a better aesthetic appearance and less treatment time as reported by Keser [65]. Piezocision is a promising tooth acceleration technique because of its various advantages on the periodontal, aesthetic, and orthodontic aspects.

### Clinical application for the future

The administration of exogenous biological molecules to accelerate tooth movement during orthodontic treatments has been intensively tested on animal experiments. However, clinical trials on humans are limited since they must be administered occasionally by local injections that can be painful and cause discomfort to the patients avoiding systemic applications, plus their side effect was not tested for long periods of time. However, administration of certain molecules has shown promising results; for example, cytokine, PTH, vitamin D, and RANKL/RANK/OPG system play an important role in bone remodeling and tooth movement. Human relaxin does not accelerate tooth movement in rats, but increases tooth mobility by decreasing the organization and mechanical strength of the PDL. However, a lot of these mechanisms are not fully understood and the dose-dependent mechanisms should also be further investigated.

In the physical approach, the low level laser therapy is the most promising method; however, contradictory results were shown. This is due to the different energies, duration, and experimental design. Furthermore, most of these experiments were done in only few weeks, which is a very short time to notice any side effects.

The surgical approach is the most clinically used and most tested with known predictions and stable results. However, it is invasive, aggressive, and costly, and patients are not open to the ideas involving surgery unless it is the only option that is needed to have a good occlusion. Piezocision technique is one of the newest techniques in accelerating tooth movement, and it has good clinical outcome and is considered the least invasive in the surgical approach.

## Conclusions

In general, all these techniques had draw backs and uncertainties that made them not commonly used clinically. However, there has been a rapid increase in the interest levels of product companies to enhance the effects of biology in orthodontics. These new approaches have the potential to be the next frontier for orthodontics and its resources.
